# Largazole Pharmacokinetics in Rats by LC-MS/MS

**DOI:** 10.3390/md12031623

**Published:** 2014-03-20

**Authors:** Mingming Yu, Lilibeth A. Salvador, Sherwin K. B. Sy, Yufei Tang, Ravi S. P. Singh, Qi-Yin Chen, Yanxia Liu, Jiyong Hong, Hartmut Derendorf, Hendrik Luesch

**Affiliations:** 1 Department of Pharmaceutics, College of Pharmacy, University of Florida, Gainesville, FL 32610, USA; E-Mails: yumingming4916@gmail.com (M.Y.); sherwin.sy@ufl.edu (S.K.B.S.); yufei@cop.ufl.edu (Y.T.); ravi.singh@ufl.edu (R.S.P.S.); hartmut@cop.ufl.edu (H.D.); 2 Department of Medicinal Chemistry, College of Pharmacy, University of Florida, Gainesville, FL 32610, USA; E-Mails: lasalvador@ufl.edu (L.A.S.); chenqyufl@ufl.edu (Q.-Y.C.); liuanka@ufl.edu (Y.L.); 3 Center for Natural Products, Drug Discovery and Development (CNPD3), University of Florida, Gainesville, FL 32610, USA; 4 Department of Chemistry, Duke University, Durham, NC 27708, USA; E-Mail: jiyong.hong@duke.edu; 5 Key Laboratory of Marine Drugs, Ministry of Education of China, School of Medicine and Pharmacy, Ocean University of China, Qingdao 266003, China; 6 Marine Science Institute, College of Science, University of the Philippines, Diliman, Quezon City, 1100, Philippines

**Keywords:** largazole, LC-MS/MS, pharmacokinetics, protein binding

## Abstract

A highly sensitive and specific LC-MS/MS method for the quantitation of largazole thiol, the active species of the marine-derived preclinical histone deacetylase inhibitor, largazole (prodrug), was developed and validated. Largazole thiol was extracted with ethyl acetate from human or rat plasma along with the internal standard, harmine. Samples were separated on an Onyx Monolithic C18 column by a stepwise gradient elution with 0.1% formic acid in methanol and 0.1% aqueous formic acid employing multiple reaction monitoring (MRM) detection. Linear calibration curves were obtained in the range of 12.5–400 ng/mL with 200 µL of human plasma. The overall intra-day precision was from 3.87% to 12.6%, and the inter-day precision was from 7.12% to 9.8%. The accuracy at low, medium and high concentrations ranged from 101.55% to 105.84%. Plasma protein bindings of largazole thiol in human and rat plasma as determined by an ultrafiltration method were 90.13% and 77.14%, respectively. Plasma drug concentrations were measured by this LC-MS/MS method. The pharmacokinetics of largazole thiol in rats was studied following i.v. administration at 10 mg/kg and found to follow a two-compartment model. Largazole thiol was rapidly eliminated from systemic circulation within 2 h. The established LC-MS/MS method is suitable for the analysis of largazole thiol in human plasma, as well.

## 1. Introduction

**Figure 1:**
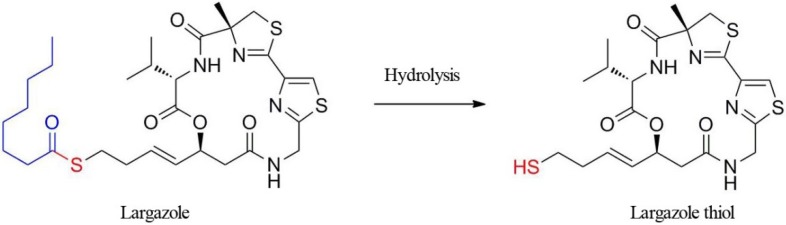
The structure of the prodrug, largazole, and hydrolytic activation to liberate largazole thiol.

The cyclic depsipeptide, largazole, discovered from a marine cyanobacterium, was first described in 2008 as a potential anticancer agent with novel chemical scaffolding and selectivity for cancer cells over non-transformed cells [1]. This activity combined with the unusual structural features has triggered the synthesis of this compound and rigorous biological evaluation. Upon completion of our first synthesis, we and, subsequently, others demonstrated that largazole is a prodrug for the potent histone deacetylase (HDAC) inhibitor [2,3], largazole thiol, which possesses selectivity for Class I HDAC isoforms, especially HDACs 1, 2 and 3, with subnanomolar potency [3,4,5]. The largazole discovery has triggered numerous additional total syntheses [6,7,8,9,10,11,12,13,14]. Largazole thiol is liberated by protein-assisted hydrolysis when exposed to cellular or plasma proteins (Figure 1) [2]. Molecular docking studies using an HDAC1 homology model and the HDAC8-largazole thiol X-ray co-crystal structure indicated that the side chain containing the thiol group chelates Zn^2+^ in the active site of HDACs, and the macrocycle interacts with divergent regions of HDACs to give pronounced selectivity for Class I isoforms, rather than broadly inhibiting the eleven Zn^2+^-dependent isoforms [2,15].

HDAC inhibitors comprise structurally diverse compounds that are used as targeted anticancer agents, since Class I HDACs, such as HDAC1 and HDAC3, are overexpressed in various cancers and are associated with cellular proliferation [16,17,18,19]. SAHA (vorinostat) and FK228 (romidepsin) are the first- and second-generation HDAC inhibitors to become commercially available. Both drugs have been approved for cutaneous T-cell lymphoma (CTCL) [16,20]. FK228 has improved selectivity for Class I HDACs with an *in vitro* profile that is similar to that of largazole [4]. These two compounds are prodrugs that liberate a thiol warhead upon metabolic activation, either by thioester hydrolysis (largazole) or by disulfide reduction (FK228). Largazole shows slightly higher potency than these two HDAC inhibitors [3,4].

We have initially characterized largazole’s activity against highly susceptible colon cancer cells, where the compound modulates gene expression, inducing cell cycle inhibitors, such as p21, and downregulating cancer-associated receptor tyrosine kinases, resulting in concentration-dependent cell cycle arrest and apoptosis [2]. Largazole also showed efficacy in a colon tumor xenograft mouse model, retarding HCT116 tumor growth without acute toxicity up to the highest dose tested (50 mg/kg) via intraperitoneal (i.p.) administration. The therapeutic dose of 5 mg/kg/day was chosen based on biochemical markers, *viz*. the stimulation of histone hyperacetylation in the tumor, which is indicative of HDAC inhibition [2]. In another *in vivo* study using orthotopic tumors derived from highly invasive MDA-MB-231 cells as a model of triple negative breast cancer, a dose of 10 mg/kg/day of largazole cooperated with dexamethasone to induce E-cadherin localization to the plasma membrane, consequently reducing the invasiveness by mediating cell-cell contacts [21]. This effect of largazole was attributed to non-histone posttranslational effects on the E-cadherin complex, since largazole increased the association of E-cadherin with γ-catenin. In addition to its *in vivo* anticancer and anti-invasive properties, largazole showed bone-forming activity in mouse and rabbit *in vivo* models [22], and it was also demonstrated that 5 mg/kg/day of largazole decreased liver fibrosis, extending the utility of largazole beyond cancer treatment [23].

The goal of the present study was to develop a validated specific LC-MS/MS analytical method for largazole, to evaluate its protein binding and to determine the pharmacokinetic property of largazole thiol in rats using this methodology.

## 2. Results

### 2.1. Assay Validation

#### 2.1.1. Specificity and Selectivity

**Figure 2:**
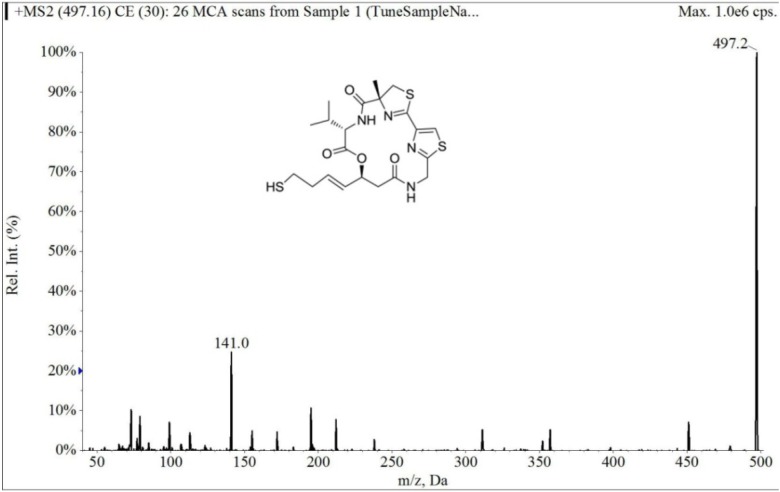
Collision-induced dissociation mass spectrum of largazole thiol.

**Figure 3:**
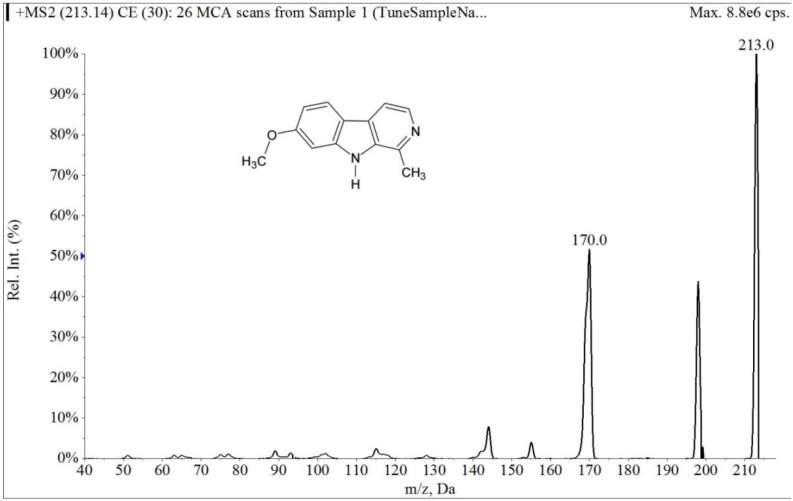
Collision-induced dissociation mass spectrum of harmine.

**Figure 4:**
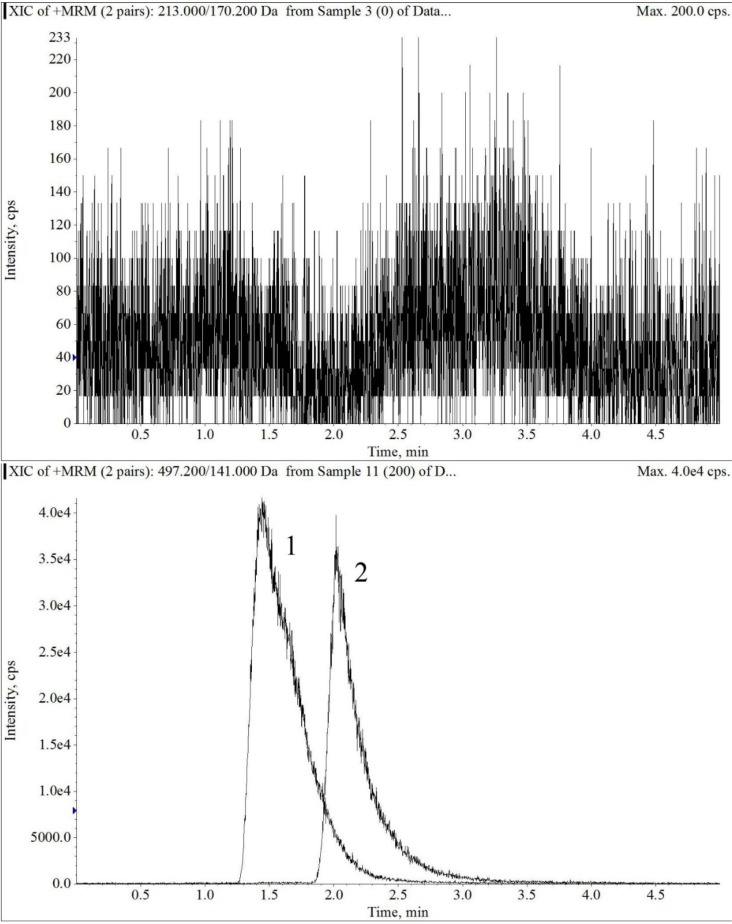
The extracted ion chromatograms of blank plasma (upper panel), largazole thiol (lower Panel 2) at 200 ng/mL and the internal standard (lower panel 1) in plasma under multiple reaction monitoring (MRM) electrospray LC-MS/MS conditions.

The electrospray mass spectrum of largazole thiol and harmine are shown in Figure 2 and Figure 3. The overall chromatographic run time was completed within 5 min with largazole thiol *t*_R_ at 2.1 min and the internal standard, harmine, at *t*_R_ of 1.5 min, as shown in Figure 4. There were no significant interfering peaks at the retention times of largazole thiol and internal standard.

#### 2.1.2. Linearity and Lower Limits of Quantification

The calculated detector response of the largazole thiol:harmine ratio *versus* the nominal concentration displayed a linear relationship in the tested range of 12.5–400 ng/mL. The correlation coefficients of 0.9989 ± 0.0008 (range: 0.9974–0.9997) were obtained for human plasma. The lower limit of quantification of 12.5 ng/mL was measured at 12.43 ± 0.91 (mean ± S.D.) from six samples with a relative standard deviation of 7.33% and a deviation of 0.56% from the nominal concentration. 

#### 2.1.3. Accuracy and Precision

Table 1 and Table 2 show a summary of intra- and inter-day precision and accuracy for largazole thiol in human plasma. The intra-day precision (R.S.D.) of largazole thiol for 37.5 ng/mL ranged from 5.12% to 10.00%; 75 ng/mL was between 8.35% and 12.56%, and 300 ng/mL was from 3.87% to 8.36%. The inter-day precision of largazole thiol ranged from 7.12% to 9.77%, and the accuracy was from 101.55% to 105.84%. 

**Table 1:** Intra-day precision and accuracy of largazole thiol in human plasma (*n* = 6).

	Nominal Concentration (ng/mL)	Measured (mean ± S.D.)	Precision R.S.D. (%)	Accuracy Deviation (%)
Day 1	37.5	39.38 ± 3.94	10.00	5.01
	75	73.58 ± 6.14	8.35	−1.89
	300	319.80 ± 26.74	8.36	6.60
Day 2	37.5	38.65 ± 2.05	5.12	3.01
	75	71.38 ± 6.24	8.75	−4.83
	300	333.00 ± 15.44	4.64	11.00
Day 3	37.5	35.78 ± 2.55	7.12	2.08
	75	76.16 ± 9.57	12.56	1.55
	300	299.80 ± 11.61	3.87	0.067

**Table 2:** Inter-day precision and accuracy of largazole thiol in human plasma (*n* = 18).

Nominal Concentration (ng/mL)	Measured (mean ± S.D.)	Precision R.S.D. (%)	Accuracy Deviation (%)
37.5	37.85 ± 3.13	8.23	2.27
75	76.16 ± 7.19	9.77	1.55
300	317.53 ± 22.59	7.12	5.84

The recovery for largazole thiol from 37.5 to 300 ng/mL using Equation (1), listed in Table 3, indicates an overall mean recovery of 81.65%. The short-term stability test showed that the differences were ≤11.73%, which is well within the 20% R.S.D. specified in the FDA guideline for industrial bioanalytical method validation (Table 4). Plasma samples were stable for three freeze and thaw cycles (Table 4).

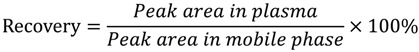
(1)

**Table 3:** Recovery of largazole thiol in human plasma (*n* = 6).

Concentration (ng/mL)	Recovery (%)
37.5	77.09
75	81.71
300	86.14

**Table 4:** Stability of largazole thiol in human plasma (*n* = 3).

Sample Condition	Nominal Concentration (ng/mL)	Measured (mean)	DEV (%)
4 h at room temperature	37.5	41.90	11.73
	300	320.33	6.78
Freeze/thaw cycle no. 1	37.5	39.70	5.87
	300	335.67	11.89
Freeze/thaw cycle no. 2	37.5	41.13	9.68
	300	298.33	−0.56
Freeze/thaw cycle no. 3	37.5	40.40	7.73
	300	315.67	5.22

#### 2.1.4. Matrix Effect

Table 5 shows the matrix effect of largazole thiol in human plasma at three concentrations, including 37.5, 75 and 300 ng/mL. The measure of matrix effect can be termed as the matrix factor (MF) and evaluated as a ratio of the analyte peak area in the presence of matrix ions (QC in the extracts of blank plasma) to the analyte peak area in the absence of matrix ions (QC in the mobile phase) using Equation (2). The overall mean matrix effect based on this equation is 72.89%.

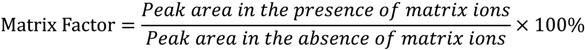
(2)

**Table 5:** Matrix effect of plasma for largazole thiol (*n* = 6).

Nominal Concentration (ng/mL)	Matrix Effect (%)
37.5	74.21
75	72.17
300	72.30

#### 2.1.5. Quality Control Samples of Rat Plasma

There is no significant difference between human and rat plasma using the analytical method. Table 6 lists quality control samples of rat plasma using the calibration curve of human plasma. The deviations were less than 10% at the three concentrations tested.

**Table 6:** Quality control samples of rat plasma (*n* = 6).

Nominal Concentration (ng/mL)	Measured	DEV (%)
37.5	36.18	−3.52
75	76.14	1.52
300	323.40	7.8

### 2.2. Protein Binding

Table 7 shows the plasma protein binding of largazole thiol in human and rat plasma, and the overall mean values of human and rat plasma are 90.13% and 77.14% using Equation (3). The non-specific binding of the compound to the membrane was 15.86%.

PB%= (1 − C_f_/(1 − NSB)/C_0_) × 100% (3)

where PB is protein binding, C_f_ is the concentration in the filtrate, C_0_ is initial concentration and NSB is non-specific binding.

**Table 7:** Plasma protein binding of largazole thiol (*n*=3).

	Drug Concentration (µg/mL)	Human (mean ± S.D.)	Rat (mean ± S.D.)
Protein binding (%)	0.5	92.52 ± 0.083	79.27 ± 1.85
	2	89.38 ± 0.94	74.39 ± 1.95
	5	88.48 ± 0.23	77.75 ± 0.86

### 2.3. Pharmacokinetics Study

#### 2.3.1. Population Pharmacokinetic Model

**Figure 5:**
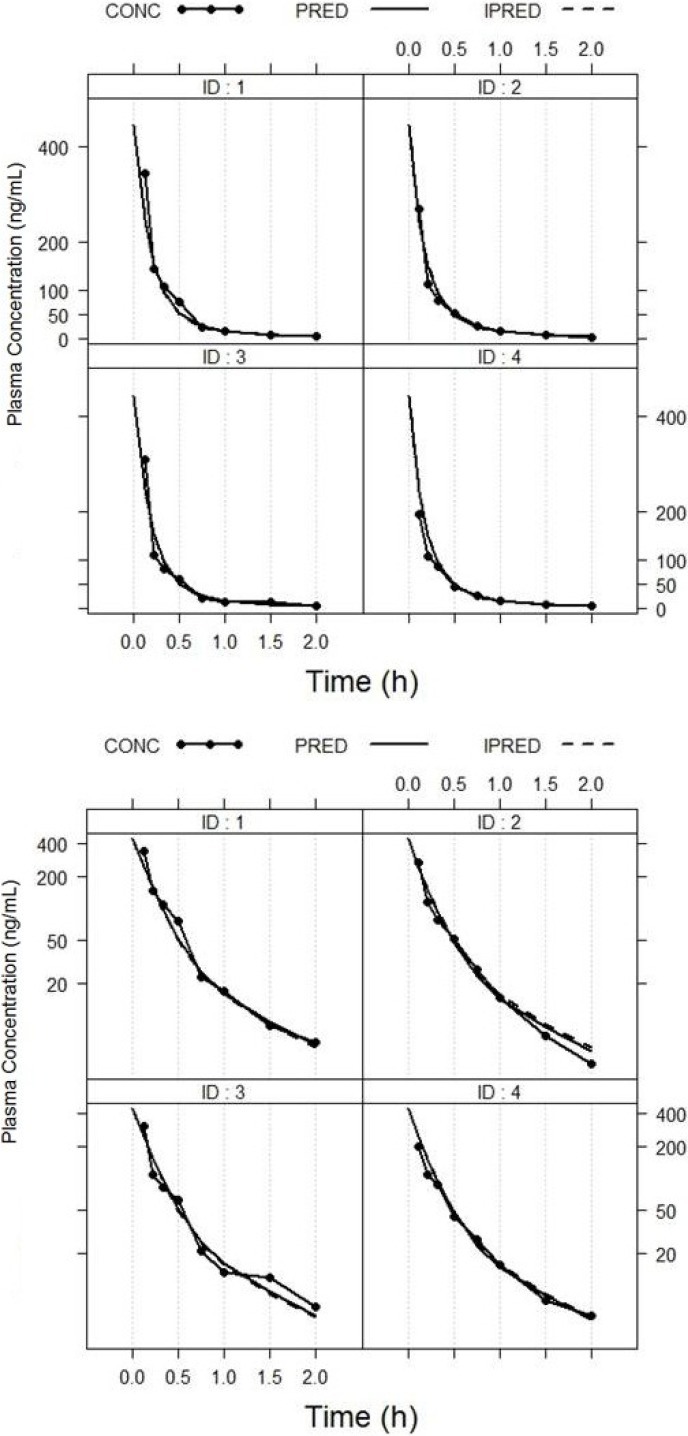
Plot of observed and individual predicted largazole thiol concentration *versus* time. PRED, population predicted fit; IPRED, individual predicted fit; CONC, observed concentration.

**Figure 6:**
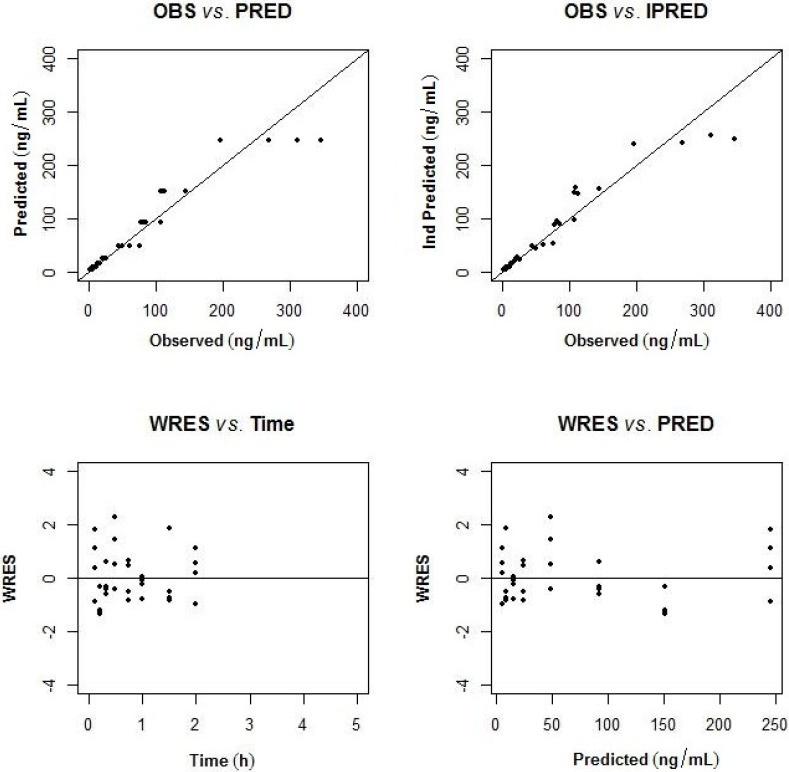
Goodness of fit plots (observed (OBS) *vs.* population predicted; observed *vs.* individual predicted concentration; weighted residuals (WRES) *vs.* time and population predicted concentration).

The pharmacokinetics of largazole thiol was analyzed in rats after administration of a 10-mg/kg intravenous bolus of largazole and samples taken between zero and 5 h. After 2 h post-dose, the drug concentrations were below the quantifiable limit. The population pharmacokinetic model was developed using a non-linear mixed-effects modeling approach. A two-compartment model best describes the disposition of largazole in rats. The pharmacokinetics parameters of largazole thiol in rats are detailed in Table 8. The calculations assume a complete conversion of largazole to its thiol. The individual observed and predicted concentration-time profiles are depicted in Figure 5. The model diagnostics in Figure 6 showed good agreement between the observed data and the model prediction. The plots of the observed (OBS) concentrations *vs.* the model predicted (PRED) concentrations and OBS *vs.* individual predicted (IPRED) concentrations are shown in the top panel of Figure 6. The weighted residuals (WRES) *vs.* time and WRES *vs.* PRED plots at the bottom panel of Figure 6 show that most of the data lies within two units from the zero-ordinate.

**Table 8:** Population pharmacokinetic model parameters of the final model.

Parameter	Mean	%SE
Structural model parameters		
Clearance (CL) (L/h/kg)	89.1	8.2
Volume of central compartment (V_c_) (L/kg)	21.8	14.5
Inter compartment clearance (Q) (L/h/kg)	26.5	19.6
Volume of peripheral compartment (V_p_) (L/kg)	17.4	14.4
Interindividual variability		
%CV of CL (ωCL)	6.06	1.23
%CV of V_c_ (ωV)	31.6	FIX
Residual variability		
Proportional residual error	−0.208	11.9
OFV	168.8	

#### 2.3.2. Non-Compartmental Analysis

The non-compartmental pharmacokinetic analysis was utilized to obtain the steady-state pharmacokinetic parameters of each individual’s concentration-time profile, using WinNonLin version 6.3 (Pharsight Corp., Sunnyvale, CA, USA). Table 9 lists the summary statistics for the pharmacokinetic parameters on the observed concentration-time profiles. 

**Table 9:** Summary statistics for the pharmacokinetic parameters of largazole.

Parameters	Mean ± S.D.
Systemic clearance, CL_obs (L/h/kg)	75.6 ± 17.9
Volume based on terminal phase, Vz_obs_ (L/kg)	54.5 ± 12.1
Volume at steady-state, Vss_obs_ (L/kg)	26.7 ± 10.9
Extrapolated zero-time concentration, C_0_ (ng/L)	804.5 ± 296.5
Area under the curve to last measurable time, AUC_last_ (h·µg/L)	133.7 ± 29.1
Area under the curve to infinity, AUCINF_obs_ (h·µg/L)	137.5 ± 29.6
Mean residence time to infinity, MRTINF_obs_ (h)	0.34 ± 0.06
Peak drug concentration, C_max_ (ng/L)	280.3 ± 63.6
Last measurable drug concentration, C_last_ (ng/L)	5.2 ± 1.2
Area under the moment curve to last time point, AUMC_last_ (h·h·µg/L)	36.0 ± 4.0
Area under the moment curve to infinity, AUMCINF_obs_ (h·h·µg/L)	46.4 ± 6.7
Half-life, *t*_1/2_ (h)	0.50 ± 0.07

## 3. Discussion

HDAC inhibitors are already available on the market, but one main concern is the metabolic stability, which consequently affects the *in vivo* activity [16]. The prodrug largazole is rapidly converted in mouse serum to the active species largazole thiol by a protein-assisted mechanism [2]. Largazole thiol may subsequently undergo reversible protein adduct formation via thioester or disulfide linkages. This trans-protection strategy has been hypothesized to potentially assist with the delivery to the site of action via carrier proteins [2]. Since largazole was demonstrated in *in vitro* experiments to be rapidly converted to the bioactive species, largazole thiol, we focused on developing an assay to detect the relevant biotransformation product largazole thiol with a relatively short analytical time [23]. The validation results showed that the accuracy and precision of this method are 5.12% to 12.56% and 101.55% to 105.84% respectively, which meet the requirement of the FDA for bioanalytical method validation. The stability test showed that the differences were ≤11.73%, which is well within 20% R.S.D., specified in the FDA guideline for industrial bioanalytical method validation. Therefore, the analytical method using LC-MS/MS was shown to be highly sensitive, specific and suitably applicable to the pharmacokinetics study of largazole in rats. In our studies, there was no significant difference between quality control samples of rat and human plasma using the calibration curve in human plasma. Therefore, the assay method and extraction procedure of largazole thiol can be extrapolated to human plasma.

Because plasma protein binding has a significant impact on the drug’s pharmacokinetics, pharmacodynamics and drug-drug interactions, the plasma protein binding of largazole thiol was evaluated using ultrafiltration in both human and rat plasma. Plasma protein binding of largazole thiol in human and rat plasma was 90.13% and 77.14%, suggesting that the free drug component of largazole thiol is 10% and 23%, respectively. The plasma protein binding of largazole thiol is slightly lower than romidepsin, with the latter being highly bound to human plasma (92%–94%) over a concentration of 50–1000 ng/mL [24,25]. As in the case of romidepsin, the plasma protein binding of largazole thiol was also independent of the concentration. In contrast, the relatively low molecular weight hydroxamate-based, vorinostat, showed lower plasma protein binding compared to the depsipeptide-based inhibitors, with only 71.3% bound to human plasma [26]. Despite the high plasma protein binding of depsipeptide-based HDAC inhibitors, the highly potent activity of these compounds, with subnanomolar IC_50_, against the cancer-relevant Class I HDACs allow for successful inhibition [4]. Largazole has low-nanomolar cellular activity, while vorinostat (SAHA) has only micromolar cellular activity [2]. 

After a single intravenous bolus administration of 10 mg/kg largazole in rats, the plasma largazole thiol concentration rapidly decreased from about 300 ng/mL to less than 10 ng/mL within an hour post-administration and was undetectable after two hours. A biphasic concentration-time profile observed in all rats was best described by a two-compartment pharmacokinetic model [27]. The volume of distribution and the clearance based on the non-compartmental analysis were consistent with the parameter values obtained from population pharmacokinetic analysis. Furthermore, largazole thiol has a similar volume of distribution (Vdss, 26.7 L/kg) to the approved HDAC inhibitor, FK228 (Vdss, 22.3 L/kg) [28]. The clearance of largazole thiol from systemic circulation was rapid, at approximately 89 L/h/kg. The clearance of largazole thiol is about two-fold greater compared to romidepsin. Vorinostat, on the other hand, has lower clearance and Vdss, 3.3 L/h/kg and 0.6 L/kg, respectively [26]. In spite of the rapid and extensive biotransformation of romidepsin, several of the romidepsin metabolites still possess the relevant thiol or intact disulfide moiety, suggesting that these metabolites can still be converted to the bioactive redFK228 and inhibit HDACs [24,25,26]. Biotransformation of vorinostat, on the other hand, converts it to the inactive *O*-glucuronide conjugate and 4-anilino-oxobutanic acid [26]. Preserving the warhead moiety following biotransformation is thus also critical to the observed functional response following HDAC inhibitor treatment. For example, the epoxide-based HDAC inhibitors, such as trapoxins have a relatively short *t*_1/2_ (<5.0 min), and biotransformation primarily occurs in the “warhead” epoxide moiety, thus rendering the biotransformation products inactive and yielding no significant *in vivo* activity [16,29].

The rapid clearance of largazole thiol may indicate its rapid tissue distribution and/or biotransformation. Largazole, because of its highly decorated macrocycle, like romidepsin, may also be metabolized outside of the thiol moiety and possibly non-detrimental to biological activity. As demonstrated for other thiol based drugs, including romidepsin [24] and captopril [30], the formation of mixed disulfides with glutathione or cysteine, as well as homodimers are also observed biotransformation products; we hypothesized the same for largazole. The biotransformation of largazole thiol will be presented in future correspondences.

## 4. Experimental Section

### 4.1. Chemicals and Reagents

Largazole and largazole thiol were synthesized according to the procedure by Ying *et al.* [5]. Harmine was purchased from Sigma-Aldrich (St. Louis, MO, USA). Methanol (HPLC grade), formic acid (HPLC grade) and ethyl acetate were purchased from Fisher Scientific (Pittsburgh, PA, USA). Isoflurane was provided by Butler Animal Health Supply (Dublin, OH, USA).

### 4.2. Instrumentation

The chromatographic system consisted of a PerkinElmer series 200 autosampler, a PerkinElmer series 200 pumps and an API 4000 mass spectrometer (AB Applied Biosystems, Framingham, MA, USA). 

#### LC-MS/MS System

The column was an Onyx Monolithic C18 (3.0 × 100 mm, Phenomenex, Torrance, CA, USA). The column temperature was kept at room temperature. The mobile phase consisted of solvent, 0.1% formic acid in MeOH (Solvent A) and 0.1% aqueous formic acid (Solvent B). With a flow rate of 0.5 mL/min and the detection mode by electrospray ionization-MS in positive ion mode (multiple reaction monitoring (MRM) scan), a stepwise gradient elution was used, starting at 60% A and 40% B, then increasing to 83% of Solvent A at 4 min and then decreasing to 60% of Solvent A at 5 min. The parameters were optimized before analysis by using direct syringe infusion. The retention times (*t*_R_, min; MRM ion pair) of the largazole thiol and internal standard are as follows: largazole thiol (2.1 min; *m/z* 497.2 → *m/z* 141.0); harmine (1.5 min; *m/z* 213.0 → *m/z* 170.2).

### 4.3. Sample Extraction and Preparation

A 1-mg/mL largazole thiol stock solution was prepared in methanol. Aliquots of this solution were diluted with the mobile phase to set a series of standards with concentrations of 12.5, 25, 50, 100, 200 and 400 ng/mL. Standard and QC samples were prepared by spiking 190 µL of blank human plasma in centrifuge tubes with 10 µL of the appropriate largazole thiol standard solution for a total volume of 200 µL. After shaking for 10 s, 1000 µL of ethyl acetate with 10 ng/mL internal standard were added to each tube, shaken for 20 min and centrifuged for 6 min at 8000 rpm. The clear supernatant was transferred and evaporated. The residue was reconstituted in 200 µL of methanol/0.1% aqueous formic acid (60:40, v/v), and shaken for 10 s. A 50 µL-aliquot of this solution was then injected for HPLC-MS analysis.

### 4.4. Assay Validation

For the calibration and regression, the non-zero point calibration curve (12.5, 25, 50, 100, 200, 400 ng/mL) was calculated by regressing the peak ratio of largazole thiol to the internal standard. Six samples of 12.5 ng/mL largazole thiol in plasma were prepared for evaluation of LLOQ; 12.5 ng/mL is the lowest concentration used for the calibration curve. Three sets of quality control samples (37.5, 75, 300 ng/mL) were prepared to assess the intra- and inter-day precision and accuracy of the assay [31]. QC samples of rat plasma were prepared to test the difference between human and rat plasma using the calibration curve of human plasma.

The liquid-liquid extraction recovery was evaluated by comparing the analytical results for extracted samples at three concentrations with un-extracted standards that represent 100% recovery [32]. The recovery study of largazole thiol was evaluated at three concentration levels (37.5, 75 and 300 ng/mL). 

To test the stability of largazole thiol, three replicates each of low and high concentrations were thawed at room temperature for 6 h to test the short-term stability. Samples at low and high concentrations were thawed at room temperature and refrozen at −20 °C for three cycles and then assayed. The matrix effect was studied by analyzing QC samples of largazole thiol in the mobile phase and extracts of plasma. Six samples at three concentration levels were prepared to calculate the matrix effect [33]. 

### 4.5. Plasma Protein Binding

Protein binding of largazole thiol was performed in human and rat plasma at 0.5, 2 and 5.0 µg/mL, using the ultrafiltration method [34]. Briefly, following the incubation of largazole thiol in plasma at 37 °C for 0.5 h, plasma containing the drug was loaded into the Centrifree^®^ Ultrafiltration Device (Millipore Corp., Carrigtwohill, Ireland), and the filtrate device was centrifuged at 1,800× *g* for 25 min at 37 °C. Concentrations of the total drug before centrifugation and the free drug in the filtrate were extracted, and the residues were assayed by HPLC-MS/MS. PBS was used to test for nonspecific binding.

### 4.6. Pharmacokinetics Study in Rats

Male Sprague Dawley rats (Charles River Laboratories International, Inc., Wilmington, MA, USA) weighing 240–260 g were used for the pharmacokinetic study of largazole. The pharmacokinetics of largazole was performed in rats by intravenous administration of 10 mg/kg. Largazole was formulated by dissolving in 20% ethanol, 20% DMSO, 20% PEG400 and 40% normal saline. A heparinized blood sample at 0.3–0.4 mL was collected from a sublingual vein puncture, according to the following schedule: 0, 0.12, 0.22, 0.33, 0.5, 0.75, 1, 1.5, 2, 3 and 5 h post-drug administration. The collected blood sample was placed on ice immediately, and plasma was separated from the blood sample by centrifugation at 2400× *g* for 20 min immediately after collection. Plasma samples were kept frozen at −80 °C until analysis by LC-MS/MS. The study was approved by the Institutional Animal Care and Use Committee of the University of Florida. 

### 4.7. Data Analysis

The population pharmacokinetic model was developed using a non-linear mixed-effects modeling approach [35]. The first-order conditional maximum likelihood estimation in the NONMEM program (double precision, version 7.2, ICON Development Solutions, Elliott City, MD, USA) and NM-TRAN pre-processor were used. The subroutines within NONMEM were linear mammillary models (ADVAN3 used with TRANS4 in the PREDPP library) to investigate a two-compartment, intravenous model. 

The non-compartmental pharmacokinetic analysis was utilized to obtain the steady-state pharmacokinetic parameters of each individual’s concentration-time profile, using the software, WinNonLin v5.3 (Pharsight, Mountain View, CA, USA). 

## 5. Conclusions

A highly sensitive and specific LC/MS/MS method for the quantitation of largazole thiol has been developed. This method has been validated and can be applied for the pharmacokinetics study of largazole thiol in rats. Plasma protein binding of largazole thiol in rat and human plasma was investigated. The pharmacokinetics study in rats indicated that largazole thiol conforms to a two-compartment behavior.

## Abbreviations

API: Atmospheric pressure ionization
CE: Collision energy
Cps: Counts per second
DEV: Deviation
DMSO: Dimethyl sulfoxide
FDA: United States Food and Drug Administration
FK228: Romidepsin
HDAC: Histone deacetylase
HCT116: Human colon cancer
i.v.: Intravenous
LLOQ: Lower limit of quantification
MCA: Mass cumulative acquisition
MDA-MB-231: Human breast adenocarcinoma 231
MS2: Tandem mass spectrometry
NIH: National Institutes of Health
NM-TRAN: NONMEM translator
NONMEM: Non-linear mixed effects modeling
OFV: Object function value
PB: Protein binding
PBS: Phosphate buffered saline
PEG400: Polyethylene glycol 400
PREDPP: Prediction for observation population pharmacokinetics
QC: Quality control
Rel. Int.: Relative intensity
R.S.D.: Relative standard deviation
SAHA: Suberoylanilide hydroxamic acid
SE: Stand error
TRANS4: Translator4
Vdss: Volume of distribution at steady state
XIC: Extracted-ion chromatogram
